# Longitudinal Rates of Change in Structural Parameters of Optical Coherence Tomography in Primary Angle Closure Glaucoma following Laser Iridotomy along with Peripheral Iridoplasty

**DOI:** 10.1155/2024/9978354

**Published:** 2024-02-27

**Authors:** Hyun-Kyung Cho, Changwon Kee

**Affiliations:** ^1^Department of Ophthalmology, Gyeongsang National University Changwon Hospital, Gyeongsang National University, School of Medicine, Changwon, Republic of Korea; ^2^Institute of Health Sciences, School of Medicine, Gyeongsang National University, Jinju, Republic of Korea; ^3^Department of Ophthalmology, Samsung Medical Center, Sungkyunkwan University School of Medicine, Seoul, Republic of Korea

## Abstract

**Background:**

This study aimed to investigate longitudinal rates of change (LRCs) of structural parameters from optical coherence tomography (OCT) in patients with primary angle closure glaucoma (PACG) after laser iridotomy (LI) along with laser peripheral iridoplasty (PI).

**Methods:**

Among 146 patients diagnosed with PACG, thirty-two subjects (32 eyes) who underwent LI plus PI and accomplished more than five times of reliable OCT tests were included in the current retrospective study. Retinal nerve fiber layer (RNFL) and Bruch's membrane opening-minimum rim width (BMO-MRW) were measured by spectral-domain OCT with three month interval. LRCs of global and six Garway-Heath sectors were investigated using the linear mixed-effects model which adjusted BMO area, sex, and age. Imaging of dual Scheimpflug analyzer was performed before and at 1 week after LI with PI and yearly thereafter.

**Results:**

The mean follow-up period was 32.28 ± 13.34 months with a mean number of 10.18 ± 3.33 OCT images. Baseline characteristics are as follows: age, 63 ± 7.9 years; female, 62.5%; intraocular pressure(IOP), 15.48 ± 4.79 mmHg; anterior chamber depth, 2.09 ± 0.18 mm; and mean deviation, −7.97 ± 8.48 dB. Global LRC of BMO-MRW was 0.86 ± 1.34 *μ*m/yr and RNFL was −0.64 ± 0.22 *μ*m/yr. IOP decreased significantly to 13.06 ± 2.21 mmHg (*p*=0.001) while anterior chamber volume (*p*=0.011) and mean anterior chamber angle (*p*=0.022) increased significantly after LI along with PI compared to the baseline at the final visit.

**Conclusions:**

LRC of a new parameter, BMO-MRW, and LRC of RNFL were relatively low in patients with PACG, following LI along with PI. After widening of the anterior chamber angle and decrease of IOP due to LI plus PI, PACG might show stable structural prognosis assessed by OCT.

## 1. Introduction

The prevalence of primary angle-closure glaucoma (PACG) is higher in Asians than in other ethnicities based on population-based worldwide reports [1, 2]. It is regarded that Asians are genetically and anatomically more susceptible to develop primary angle-closure glaucoma (PACG) than other ethnicities [3, 4]. Even though the pupillary block mechanism is considered to be principally attributed to PACG, additional mechanisms might be also involved in PACG [5–7]. Peripheral laser iridotomy (LI) can relieve pupillary block through creating a passage which enables aqueous to move forward to the anterior chamber from the posterior chamber [8, 9]. It is recognized as one of the primary therapeutic modalities of PACG [8, 9]. According to previous studies, some eyes of PAC that received LI showed remained closed anterior chamber angle (ACA), including in one of our previous studies [10–13]. Furthermore, the substantial part of PACG eyes which were treated with LI demonstrated aggravation of peripheral anterior synechiae (PAS) [14–16]. We previously found that LI along with peripheral iridoplasty (PI) opened the ACA at the periphery better than LI alone calculated with the iridotrabecular contact (ITC) index [17]. The ITC index is defined as a representative percentage of the entire amount of circumferential peripheral angle closure (i.e., (degree of angle closure)/360°) [18]. PI might be enable to relieve PACG arose by other mechanisms than pupillary block, for example, anterior placement of the lens and plateau iris or thick peripheral iris [11, 17].

On the basis of these results of our previous reports, we have a preference to perform PI at the time of LI on eyes of PAC spectrum. PI generates contraction of the peripheral iris and pulls the iris off the peripheral angle, subsequently opening the ACA at the periphery [19]. It has been reported that PI is a comparatively safe procedure for PACG [20, 21].

Glaucoma lead to the damage of retinal ganglion cells (RGCs) and their axons, resulting in retinal nerve fiber layer (RNFL) deficit and neuroretinal rim (NRR) thinning that can bring about visual field (VF) loss [22]. In the early stage of glaucoma, detectable structural change precedes functional VF defect at each individual aspect [23–27]. As one of structural tests, optical coherence tomography (OCT) is widely employed in clinical circumstances. It is beneficial for diagnosing glaucoma, especially at an early phase [28].

Lately, spectral-domain OCT provides a new parameter, Bruch's membrane opening-minimum rim width (BMO-MRW), in addition to peripapillary RNFL which is more generally used. BMO-MRW is defined as the shortest distance from the BMO inner opening to the internal-limiting membrane, which has been presented for the evaluation of the optic disc [29–33]. BMO-MRW provides more accurate evaluation of the NRR than conventional optic disc inspection [29–34]. Previous studies have demonstrated that BMO-MRW showed superior diagnostic ability in glaucoma to preexistent parameters of NRR [35–37].

The longitudinal rate of change (LRC) is imperative in the management of glaucoma because it provides information regarding determination of the intensity of glaucoma treatment. Ethnicity and glaucoma subtype have been reported to have important influence on the LRC [38]. However, the LRC of BMO-MRW in PACG patients has not been previously reported. Recently, LRC of BMO-MRW and RNFL in POAG patients from Korean population have been reported but not in PACG patients [39]. Moreover, the LRC of BMO-MRW or RNFL in PACG patients after LI combined with PI has not been studied yet.

Thus, the objective of this longitudinal cohort study was to investigate the LRC of BMO-MRW, a new parameter, and the LRC of conventional RNFL in PACG patients after LI combined with PI in a single ethnic group of Asians (Koreans). We aimed to investigate the structural prognosis of PACG patients after LI plus PI assessed by OCT parameters with a long-term follow-up of more than two years.

## 2. Methods

### 2.1. Ethical Statement

This retrospective, observational, cohort study was performed according to the tenets of the Declaration of Helsinki. The present study was approved by the Institutional Review Board (IRB) of Gyeongsang National University Changwon Hospital, Gyeongsang National University, School of Medicine (GNUCH-2019-09-031-005). The requirement for informed consent was waived by the IRB because of the retrospective nature of the study.

### 2.2. Subjects

Among patients diagnosed with PACG and treated with LI along with PI at the Glaucoma Clinic of Gyeongsang National University Changwon Hospital, those with more than five reliable OCT (Spectralis® OCT, Heidelberg Engineering Inc., Heidelberg, Germany) tests who met the following criteria were included for the final investigation. Subjects aged more than 18 years old were included in this study [34]. Those subjects underwent OCT imaging at the interval of 3 months. To calculate the rate of change and for the slope to be significant, at least 5 times of serial data are required for the analysis as reported by previous studies [40–43].

PACG was determined as eyes with a shallow anterior chamber (appositional contact from the trabecular meshwork (TM) to the peripheral iris of >270 degrees by gonioscopic examination) demonstrating glaucomatous optic disc damage (NRR showing a cup-to-disc ratio of 0.7 in vertical meridian or an asymmetry between eyes of 0.2 or NRR notching owing to glaucoma), showing corresponding VF loss [44]. Diagnosis and assessment of PACG were conducted by a single glaucoma specialist (H-K Cho) to consistently apply the diagnostic criteria. Solely newly diagnosed cases were included for the analysis. Chronic PACG cases with increased uncontrolled IOP demonstrating definite PAS which might further require filtering surgery were excluded.

All included subjects underwent examinations with Spectralis spectral-domain OCT, dual Scheimpflug analyzer, and standard automated perimetry (HFA model 840; Humphrey Instruments, Inc., San Leandro, CA, USA). Merely reliable visual field tests were included according to the following criteria: fixation loss <20%; a false negative rate <15%; and a false positive rate <15%. Measurement of BMO-MRW and RNFL was performed with spectral-domain OCT at 3-month intervals. Only those with adequate image quality of both BMO-MRW and RNFL tests were included.

Exclusion criteria were as follows: defective images owing to poor fixation or eyelid blinking, any history of other optic neuropathies but for glaucoma (e.g., acute ischemic optic neuritis or optic neuritis), any history of intraocular surgery except for uneventful phacoemulsification, secondary angle closure caused by other diseases (e.g., neovascular glaucoma or uveitic glaucoma), any retinal diseases resulting in retinal edema or swelling and subsequent swelling of BMO-MRW or RNFL, history of an acute angle-closure attack which could influence the thickness of RNFL or BMO-MRW, and severe stage of glaucoma defined as the mean deviation (MD) < −12 dB. In cases of binocular eyes qualified for inclusion, just single eye was chosen in a random manner.

### 2.3. Optical Coherence Tomography

Optic disc images were obtained with a spectral-domain OCT (Spectralis® OCT, Heidelberg Engineering Inc., Heidelberg, Germany) using Glaucoma Module Premium Edition by a single experienced technician. Radial B-scans of 24 in number were acquired to analyze BMO-MRW. Among three scan circle diameters (3.5, 4.1, and 4.7 mm), a scan circle diameter of 3.5 mm was chosen for peripapillary RNFL thickness measurement. Only those images that were correctly centered and accurately segmented and quality scores ≥20 were selected for this study. Images taken with OCT were aligned in FoBMO axis, that is, an individual specific axis that measures between the center of BMO and the fovea of macula. Employing this FoBMO axis could enable more correct analysis of the Garway-Heath sector considering cyclotorsion of each individual and more precise analysis compared with normative database than the existing way of using only simple clock-hour locations [29].

### 2.4. Laser Peripheral Iridotomy and Peripheral Iridoplasty

LI along with PI was performed by a single glaucoma specialist (H-K Cho) on the same day. PI was performed before LI. PI was carried out with argon laser with a spot size of 500 mm for 0.4∼0.5 seconds and a titrated power of 150∼300 mW depending on the response of individual iris [21]. Laser beams were aimed to the iris root to obtain a visible contraction of the iris at the periphery. About 24 laser burns were employed through 360° with a 2-burn space apart. Large radial vessels were avoided carefully throughout the laser procedure.

LI was conducted on the superior area of the iris (10∼2 o'clock) with an argon laser and followed by neodymium-yttrium-aluminum-garnet lasers. Pilocarpine of concentration of 2% was applied at 30 minutes prior to LI. An argon laser at the power of 600–1000 mW and a spot size of 50∼100 *μ*m was applied and, subsequently, a yttrium-aluminum-garnet laser at 2–5 mJ for 0.05 seconds.

### 2.5. Dual Scheimpflug Analyzer Imaging

Dual Scheimpflug analyzer (Galilei G4; Ziemer Ophthalmic Systems, Port, Switzerland) imaging was acquired for the analysis of ACA parameters. High resolution image was obtained before LI + PI, at 1 week after LI + PI, and yearly thereafter. Dual Scheimpflug analyzer imaging was done in advance of any contact operation or the initiation of any hypotensive glaucoma medications. All images were taken in a sitting position under constant dim light (15 lux). It is a noncontact device and is based on analyzed images from a processed rotating dual Scheimpflug and Placido disks. It compensates eye motion based on features of the iris and examines the changes of ACA metrics and the cornea.

### 2.6. Statistical Analysis

Comparisons of ACA parameters and IOP between the baseline and last visit were conducted using the Wilcoxon signed-rank test. To calculate LRC or the progression rate as a regression coefficient, that was the slope of each parameter (BMO-MRW and RNFL), we employed a generalized linear mixed-effects model including a random intercept. LRCs were calculated with the linear mixed-effects model after adjusting for age, sex, and BMO area in the global region and in each Garway-Heath sector. Comparison of the LRC of BMO-MRW and RNFL values were analyzed with a *t*-test. The percent coefficient was estimated by setting the initial intercept to 100 at time 0. Statistical significance was considered when a *p* value was less than 0.05. All statistical analyses were conducted using SAS software version 9.4 (SAS Institute, Cary, NC, USA) and SPSS software version 20.0 (SPSS, Inc., Chicago, IL, USA).

## 3. Results

### 3.1. Baseline Characteristics

Among 146 patients with PACG who were treated with LI along with PI, 44 patients with acute angle-closure attack were excluded. Among the rest of the 102 PACG patients, those who did not undergo more than five times of reliable OCT tests or whose images did not meet the eligible quality were excluded. Those who did not meet the image quality were due to poor cooperation possibly for old age and narrow palpebral fissure, which are the characteristics of PACG patients. However, those who cooperated well for image acquisition showed excellent quality score provided by Spectralis OCT, which was 28.84 ± 3.14. A total of 32 eyes of 32 patients who received LI along with PI and had more than five times of reliable OCT tests were included for the ultimate investigation.

Twenty subjects were females and 12 subjects were males (all Koreans). The mean age was 63.31 ± 7.99 years. The mean follow-up period was 32.28 ± 13.34 months with a mean number of 10.19 ± 3.34 OCT tests. Mean quality was 32.35 ± 2.78, which was excellent. The baseline FoBMO angle was −5.92 ± 3.30° and the BMO area was 2.33 ± 0.50 mm^2^.

The baseline anterior chamber depth (ACD) between corneal endothelium and anterior surface of the lens was 2.09 ± 0.19 mm, with a mean spherical equivalent (SE) of 0.88 ± 1.19 Diopter. Baseline MD was −7.97 ± 8.49 dB. Detailed baseline characteristics are demonstrated in Table 1.

### 3.2. Changes of the Angle Status and Intraocular Pressure between Initial and Final Visits

ACD increased from the baseline of 2.09 ± 0.19 mm to 2.15 ± 0.32 mm at the final visit although such increase was not statistically significant (*p*=0.136). Anterior chamber volume (ACV) and ACA increased significantly from the baseline to the final visit (*p*=0.011 and *p*=0.022, respectively). IOP decreased significantly from the baseline of 15.48 ± 4.79 mmHg to 13.06 ± 2.21 mmHg at the final visit (*p*=0.001). However, corneal white to white from temporal to nasal limbus and mean pachymetry from three zones of central, middle, and peripheral zones did not reveal any significant changes between baseline and final visit (all *p* > 0.72) (Table 2).

### 3.3. Longitudinal Rate of Change of BMO-MRW and RNFL

LRC or the rate of progression was calculated as a coefficient by the linear mixed-effects model. Global LRC of BMO-MRW was +0.803 ± 1.329 *μ*m/year. Global LRC of RNFL was −0.641 ± 0.217 *μ*m/year. Among six Garway-Heath sectors, the inferotemporal sector showed the greatest LRC in both BMO-MRW and RNFL. Inferotemporal LRC of BMO-MRW was −1.895 ± 1.908 *μ*m/year and that of RNFL was −1.229 ± 0.310 *μ*m/year. LRC of BMO-MRW in other sectors showed positive rates as that in the global region. LRCs of RNFL in all sectors showed negative rates as that in the global region. Superotemporal LRC of BMO-MRW was +1.762 ± 1.585 *μ*m/year and that of RNFL was −0.778 ± 0.220 *μ*m/year. Detailed LRC of each sector is demonstrated in Table 3.

### 3.4. Comparison of the Rate of Change between BMO-MRW and RNFL

LRCs did not demonstrate significant difference between BMO-MRW and RNFL in the global region or any of the six Garway-Heath sectors (*t*-test, all *p* > 0.05). Detailed LRCs of each sector and global region are shown in Table 4.

Since baseline values of BMO-MRW and RNFL as well as scales of these two parameters were different, LRCs were also compared in percent reduction. LRC calculated in percent reduction showed significant differences between BMO-MRW and RNFL in the global region and superotemporal and nasal sectors (*p* < 0.0296). LRC in percent reduction of BMO-MRW showed positive rates except for the inferotemporal sector, while those of RNFL showed negative rates in all sectors and global region. Global LRC of BMO-MRW in percent reduction was +0.2322 ± 0.3842%/year while that of RNFL was −0.7834 ± 0.2652%/year (*p*=0.0296). Detailed LRCs in percent reduction for the global region and six Garway-Heath sectors are shown in Table 5.

**Figure 1:**
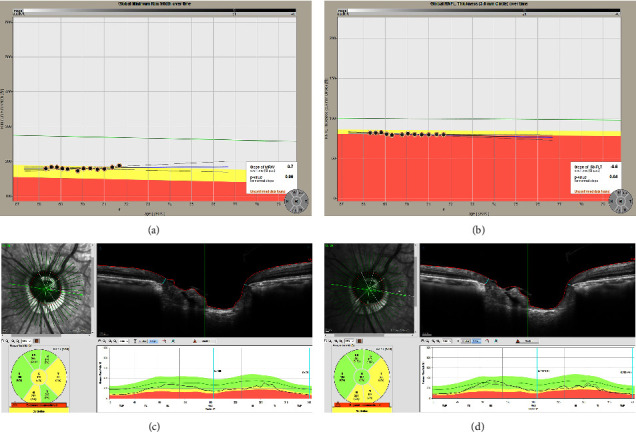
A representative case of a 72-year-old female patient with primary angle-closure glaucoma who underwent laser iridotomy (LI) combined with peripheral iridoplasty (PI). Slopes of BMO-MRW and RNFL from January 2018 to June 2021 are shown. Simple regression analysis estimating the slope of merely the global BMO-MRW (a) and the RNFL (b) by installed automated software of OCT is displayed. Note that the slope of BMO-MRW was +0.7 *μ*m/yr, while the slope of the RNFL was −0.6 *μ*m/yr. Global BMO-MRW was 179 *μ*m at the initial visit (c) and that of the last visit (d) was 187 *μ*m. Note that global BMO-MRW increased after LI combined with PI until the last visit, which was 3 years and 5 months after the first visit. ^*∗*^BMO-MRW: Bruch's membrane opening-minimum rim width, RNFL: retinal nerve fiber layer.

Figure 1 demonstrates a representative case of a 72-year-old female patient with PACG who was treated with LI along with PI. Slopes of BMO-MRW and RNFL from January 2018 to June 2021 are shown. Simple regression analysis calculating the slope of just global BMO-MRW (A) and global RNFL (B) by installed automatic software from OCT device is shown. The slope of BMO-MRW was +0.7 *μ*m/yr while that of the RNFL was −0.6 *μ*m/yr. Global BMO-MRW was 179 *μ*m at the initial visit (C) and that of the last visit (D) was 187 *μ*m. Global BMO-MRW increased after LI along with PI till the last visit, which was 3 years and 5 months after the first visit.

## 4. Discussion

In the present study, we demonstrated LRCs of BMO-MRW and RNFL in PACG patients after LI combined with PI in a single ethnic group of Asians (Koreans), which has not been reported before. During a follow-up period of more than 2.5 years, LRCs of BMO-MRW and RNFL were relatively slow. They showed a stable structural prognosis. ACA parameters revealed sustained improvement after LI plus PI even after a long period of 2.5 years. IOP also showed significant decrease at the final visit compared to that at the baseline.

In the current study, global rate of BMO-MRW was +0.857 ± 1.329 *μ*m/yr and that of RNFL was −0.641 ± 0.217 *μ*m/yr in PACG patients after LI plus PI. A previous study has reported rates of RNFL and ganglion cell complex (GCC) by OCT in PACG compared with POAG [45]. They defined progression by standard automated perimetry and divided subjects into two groups of progression and nonprogression. In their study, 59% of eyes with PACG and 57% of eyes with POAG showed progression. LRCs of RNFL (−2.95 ± 1.85 *μ*m/yr) and GCC (−3.22 ± 2.96 *μ*m/yr) were significantly higher in PACG eyes that showed progression than in POAG (−1.64 ± 2.00 *μ*m/yr (*p*=0.018) and −1.74 ± 2.05 *μ*m/yr (*p*=0.046), respectively). In PACG patients, LRC of RNFL was −2.95 ± 1.85 *μ*m/yr in the progression group (*n* = 38) and −0.29 ± 1.75 *μ*m/yr in the nonprogression group (*n* = 27), showing a significant (*p* ≤ 0.0001) difference. They performed LI on PACG patients at the beginning of the study. LRC of RNFL from the current study (−0.641 ± 0.217 *μ*m/yr) after LI combined with PI was similar to that of the nonprogression group after LI in the previous study of PACG patients. However, our results showed a relatively more stable structural prognosis assessed by OCT compared with the previous study.

In the Duke glaucoma registry study with a large population of glaucoma suspect and glaucoma patients, 29,548 OCT tests were included [46]. The LRC of global RNFL was −0.73 ± 0.80 *μ*m/year. The criterion for slow progression was defined as less than −1.0 *μ*m/year. According to this criterion, the LRC of our result of PACG (−0.641 ± 0.217 *μ*m/yr) belongs to a slow progression group. Moreover, it is similar to that of the overall rate of all included patients. Our LRC results from PACG patients after LI along with PI were not greater than the overall LRC from a large clinical population.

It was notable that the LRC of global BMO-MRW was even positive in the present study. It implies that the amount of NRR has increased during the long-term period of over 2.5 years of follow-up. Reversal of cupping is frequently observed after trabeculectomy [47]. The mechanisms underlying this cupping reversal in glaucoma of childhood has been proposed as enlargement of prelaminar tissue, contraction of scleral canal opening, and dislocation of the lamina cribrosa [48, 49]. In adult patients who underwent trabeculectomy, BMO-MRW also significantly increased after the filtering surgery and persisted for up to a year [48, 49]. Such increase of BMO-MRW was measured at an average of 7.74 *μ*m. It also correlated with IOP reduction [49]. In the present study, for over 2.5 years of the follow-up period, IOP decreased significantly from 15.48 ± 4.79 mmHg at the baseline to 13.06 ± 2.21 mmHg at the last visit (*p*=0.001). Since IOP decreased significantly after LI along with PI until the final visit after more than 2.5 years, it might have influenced the change of BMO-MRW, thus showing slight increase of BMO-MRW through the final visit in the present study. No previous study has observed PACG patients for this long after LI plus PI. No studies have found a positive rate of BMO-MRW either. However, RNFL thickness does not significantly retrieve in the reversal of the cup case. Paradoxical thinning of the RNFL has been noted on cup reversal cases in children with glaucoma [47–49]. These findings may suggest that cup reversal is not probably owing to reversal of glial remodeling or regeneration of axons [47]. In the current study, LRC of RNFL in adults also demonstrated a small negative rate, in accordance with previous observations [47–49].

Previous long-term studies evaluating ACA parameters after laser procedures included only LI without considering LI along with PI. A study from China called the Zhongshan angle-closure prevention trial followed-up these patients after LI up to 18 months [50]. It found that ACA width of eye treated with LI significantly increased at two weeks after LI, persisted constant for six months, nevertheless, significantly reduced at 18 months after LI in PAC suspects [50]. Other study conducted on PAC suspects in South Korea [51] revealed that ACA had a tendency to be shallowed after 18 months following LI in spite of the relief of pupillary block. Additional study from South Korea that followed for a long-term period of 41–54 months in PAC spectrum eyes reported that ACA widened after only LI in the pupillary block group and the thick peripheral iris group but, however, not in the exaggerated lens vault group and the plateau iris configuration group [52]. These results of other studies support the results of our previous studies [11, 17, 53] demonstrating that LI may be able to relieve only the pupillary block mechanism and that combined mechanisms besides pupillary block might also be involved in PAC, particularly in Asians where the prevalence of PACG is high [1–4]. It is considered that PI is able to relieve other mechanisms contributing to PAC than pupillary block. Thus, LI along with PI might be more beneficial than LI alone, especially in Asians, as demonstrated by previous studies of ours [11, 17, 53] and also by the current study. All parameters of ACA remained significantly widening in comparison with the baseline during more than two and a half years of follow-up in this study. Moreover, the current study is the first to include only PACG patients with a long-term period of follow-up after LI along with PI. Other previous studies included PAC spectrum eyes or PAC suspect, not just PACG. Thus, this study is meaningful to be added in the literature.

In our previous study, we calculated the LRC of BMO-MRW and RNFL in early normal tension glaucoma (NTG) patients using the same OCT [40]. The LRC of BMO-MRW was −2.06 ± 0.65 *μ*m/yr and that of RNFL was −0.96 ± 0.16 *μ*m/yr in early NTG, which showed open angle. It is noticeable that these LRCs were even faster than those investigated in the present study in PACG patients after LI combined with PI, which showed stable structural prognosis over the long-tern follow-up period. Recently, LRC of BMO-MRW and RNFL in POAG patients from Korean population has been reported but not in PACG patients [39]. They reported that the LRC of BMO-MRW POAG patients was −2.59 ± 0.24 *μ*m/yr and that of RNFL was −1.19 ± 0.09 *μ*m/yr, which were also faster than those of LRCs of PACG patients in the present study.

In the present study, we excluded patients with chronic PAS and uncontrolled IOP requiring filtering surgery. This is because filtering surgery might affect the prognosis of PACG besides the laser procedure of LI combined with PI. Elevated IOP might also affect structural prognosis of PACG assessed by OCT. Elevated IOP might have an impact on the thickness of BMO-MRW or RNFL. Moreover, the thickness of BMO-MRW or RNFL might alter after filtering surgery when IOP is dramatically reduced [47]. This inclusion of patients without any chronic PAS or elevated IOP might have affected structural prognosis in the present study, which showed a relatively favorable prognosis.

The current study is limited in several aspects. One probable limitation is the retrospective nature of this study. Only those who had completed more than five times of both BMO-MRW and RNFL OCT images with an acceptable quality were included for the analysis. Inclusions of such subjects may have a potential effect on the results of our study. However, to calculate LRC or the slope of progression, at least 5 reliable images were required. Second, this study was conducted at a tertiary hospital of a provincial national university. It was a hospital-based study and it did not have a population-based design. Hence, included subjects in the current study might not represent the whole PACG patient population. Finally, the present study had a relatively small sample size even though this longitudinal design might have limited wide inclusion. Among 146 PACG patients, only 32 eyes form 32 subjects were included to be analyzed because substantial number of patients was excluded due to inadequate number of OCT tests or poor images. Moreover, acute angle-closure cases were also excluded due to transient swelling of RNFL or BMO-MRW. Nevertheless, including 32 subjects with a follow-up for more than two and a half years might not be insufficient to evaluate LRC in a single disease of PACG to see the trend. In addition, our study included only East Asian (Korean) patients. Regarding the difference of the ACA anatomy and the prevalence of PACG in Asians [1–4], our study results might not be applicable to other ethnicities. However, considering the rarity of PACG patients in other ethnic populations, recruiting 32 PACG patients with a long-term follow-up after both LI and PI might not be easy and thus, our study has its significance in providing long-term results on structural OCT parameters along with ACA parameters after the laser procedure.

In conclusion, the LRC of BMO-MRW as a new parameter and the LRC of RNFL were relatively low in patients with PACG after LI combined with PI during a long-tern follow-up period of more than 2.5 years. After widening of ACA and decrease of IOP due to LI combined with PI, PACG might show stable structural prognosis assessed by OCT. Future studies with population-based design including large numbers and longer follow-up are required to draw definite conclusions.

## Figures and Tables

**Table 1:** Baseline characteristics of the PACG patients treated with peripheral laser iridotomy combined with iridoplasty.

Baseline characteristics	Values
Number of subjects	32 eyes (32 patients)
Mean age (year)	63.31 ± 7.99 years (52∼76)
Gender (male : female)	12 : 20
Family history of glaucoma (%)	1 (3.12%)
Mean follow-up period (months)	32.28 ± 13.34 (11∼58)
Mean number of OCT tests	10.19 ± 3.34 (5∼16)
Mean quality	32.35 ± 2.78 (26∼38)
Baseline FoBMO angle (°)	−5.92 ± 3.30 (−13.39∼2.46)
Baseline BMO area (mm^2^)	2.33 ± 0.50 (1.101∼3.591)
Baseline BMO-MRW (*μ*m)	180.53 ± 61.59 (77.16∼358.09)
Baseline RNFL (*μ*m)	74.81 ± 24.45 (38∼134)
ACD (mm)	2.09 ± 0.19 (1.66∼2.38)
SE (D)	0.88 ± 1.19 (−1.375∼4.125)
CCT (*μ*m)	543.86 ± 41.40 (483∼625)
MD (dB)	−7.97 ± 8.49 (−16.46∼0.66)
PSD (dB)	6.20 ± 4.57 (2.02∼17.73)
VFI (%)	77.94 ± 26.94 (28∼99)

*Note*. Values represent the mean ± mean deviation. PACG, primary angle-closure glaucoma; OCT, optical coherence tomography; BMO-MRW, Bruch's membrane opening-minimum rim width; RNFL, retinal nerve fiber layer; ACD, anterior chamber depth (from corneal endothelium to anterior lens surface); SE, spherical equivalent; D, diopters; CCT, central corneal thickness; MD, mean deviation; PSD, pattern standard deviation; VFI, visual field index.

**Table 2:** Changes of the angle status and IOP in subjects with PACG after peripheral laser iridotomy combined with iridoplasty.

Characteristics	Baseline	Final visit	*p* value
ACD (mm)	2.09 ± 0.19	2.15 ± 0.32	0.136
ACV (mm)	78.32 ± 11.49	83.04 ± 11.16	**0.011**
ACA (mm)	26.86 ± 2.53	28.82 ± 4.64	**0.022**
IOP (mmHg)	15.48 ± 4.79	13.06 ± 2.21	**0.001**
WTW N-T (mm)	11.48 ± 0.32	11.43 ± 0.34	0.089
Pachy central (*μ*m)	559.03 ± 35.87	554.50 ± 38.61	0.072
Pachy middle (*μ*m)	609.23 ± 35.11	603.77 ± 40.66	0.117
Pachy peripheral (*μ*m)	685.65 ± 48.64	687.13 ± 79.19	0.262

*Note*. Values represent the mean ± mean deviation. PACG, primary angle-closure glaucoma; ACD, anterior chamber depth (from corneal endothelium to anterior lens surface); ACV, anterior chamber volume; ACA, mean anterior chamber angle; IOP, intraocular pressure; WTW N-T, white to white from nasal to temporal limbus; Pachy mean pachymetry from central (0.0–4.0 mm), middle (4.0–7.0 mm), and peripheral zone (7.0–10.0 mm). Wilcoxon signed-rank test; bold font indicates significant *p* values (*p* < 0.05).

**Table 3:** Progression rate of BMO-MRW and RNFL per year in subjects with PACG.

Outcomes		Baseline value/progression rate	Standard error	95% CI		*p* value
				Lower	Upper	
BMO area	Baseline (mm^2^)	2.33	0.50			
	Coefficient (mm^2^/yr)	−0.002	0.002	−0.006	0.003	0.4543

BMO-MRW global	Baseline (*μ*m)	180.53	61.59			
	Coefficient (*μ*m/yr)	0.803	1.329	−1.815	3.420	0.5465

RNFL global	Baseline (*μ*m)	74.81	24.45			
	Coefficient (*μ*m/yr)	−0.641	0.217	−1.069	−0.213	**0.0035**

BMO-MRW T	Baseline (*μ*m)	133.15	43.79			
	Coefficient (*μ*m/yr)	0.094	1.168	−2.205	2.394	0.9357

RNFL T	Baseline (*μ*m)	61.15	17.46			
	Coefficient (*μ*m/yr)	−0.473	0.122	−0.712	−0.233	**0.0001**

BMO-MRW TS	Baseline (*μ*m)	169.65	77.07			
	Coefficient (*μ*m/yr)	1.762	1.585	−1.360	4.883	0.2675

RNFL TS	Baseline (*μ*m)	97.00	40.31			
	Coefficient (*μ*m/yr)	−0.778	0.220	−1.211	−0.345	**0.0005**

BMO-MRW TI	Baseline (*μ*m)	179.84	76.61			
	Coefficient (*μ*m/yr)	−1.895	1.908	−5.651	1.861	0.3214

RNFL TI	Baseline (*μ*m)	94.53	45.23			
	Coefficient (*μ*m/yr)	−1.229	0.310	−1.839	−0.620	**<0.0001**

BMO-MRW N	Baseline (*μ*m)	197.67	80.66			
	Coefficient (*μ*m/yr)	2.073	1.726	−1.326	5.472	0.2309

RNFL N	Baseline (*μ*m)	61.03	22.11			
	Coefficient (*μ*m/yr)	−0.489	0.227	−0.935	−0.042	**0.0321**

BMO-MRW NS	Baseline (*μ*m)	207.85	78.15			
	Coefficient (*μ*m/yr)	1.128	1.507	−1.839	4.095	0.4547

RNFL NS	Baseline (*μ*m)	92.50	41.60			
	Coefficient (*μ*m/yr)	−0.773	0.424	−1.608	0.061	0.0693

BMO-MRW NI	Baseline (*μ*m)	224.16	74.99			
	Coefficient (*μ*m/yr)	0.256	1.933	−3.551	4.063	0.8948

RNFL NI	Baseline (*μ*m)	83.96	32.30			
	Coefficient (*μ*m/yr)	−0.889	0.465	−1.804	0.027	0.0570

*Note*. *p* value by generalized the linear mixed model including random intercept for subjects after adjusting for age, sex, and BMO area. The progression rate is calculated by coefficient by time per year (*μ*m/yr). Bold font indicates significant *p* values (*p* < 0.05) of the estimated slope (progression rate). BMO-MRW, Bruch's membrane opening-minimum rim width; RNFL, retinal nerve fiber layer; PACG, primary angle closure glaucoma; G, global; T, temporal; TS, superotemporal; NS, superonasal; N, nasal; NI, inferonasal; TI, inferotemporal.

**Table 4:** Comparison of progression rates between BMO-MRW and RNFL in each sector.

Comparing coefficients by sector (raw data)		
Sectors	Z-score	*p* value
Global	1.0723	0.2836
T	0.4828	0.6292
TS	1.5873	0.1124
TI	−0.3445	0.7304
N	1.4717	0.1411
NS	1.2143	0.2246
NI	0.5759	0.5647

*Note*. *p* value by the *t*-test. BMO-MRW, Bruch's membrane opening-minimum rim width; RNFL, retinal nerve fiber layer; G, global; T, temporal; TS, superotemporal; NS, superonasal; N, nasal; NI, inferonasal; TI, inferotemporal.

**Table 5:** Comparison of percent reduction in progression rates between BMO-MRW and RNFL.

Coefficients by sector (percent reduction)				
Sectors	Percent coefficient, BMO-MRW	Percent coefficient, RNFL	Z-score	*p* value
Global	0.2322 ± 0.3842	−0.7834 ± 0.2652	2.1752	**0.0296**
T	0.0430 ± 0.5337	−0.8259 ± 0.2130	1.5120	0.1305
TS	0.4755 ± 0.4277	−0.5758 ± 0.1628	2.2970	**0.0216**
TI	−0.7895 ± 0.7949	−1.8629 ± 0.4699	1.1625	0.2450
N	0.4535 ± 0.3776	−0.8020 ± 0.3723	2.3677	**0.0179**
NS	0.2832 ± 0.3784	−0.5881 ± 0.3226	1.7524	0.0797
NI	0.0703 ± 0.5305	−0.8074 ± 0.4223	1.2943	0.1956

*Note*. *p* value by the *t*-test. Bold font indicates significant *p* values (*p* < 0.05). BMO-MRW, Bruch's membrane opening-minimum rim width; RNFL, retinal nerve fiber layer; G, global; T, temporal; TS, superotemporal; NS, superonasal; N, nasal; NI, inferonasal; TI, inferotemporal.

## Data Availability

The data used to support the findings of this study are available from the corresponding author upon reasonable request.
